# TRPA1 Is Functionally Expressed Primarily by IB4-Binding, Non-Peptidergic Mouse and Rat Sensory Neurons

**DOI:** 10.1371/journal.pone.0047988

**Published:** 2012-10-25

**Authors:** Marie E. Barabas, Elena A. Kossyreva, Cheryl L. Stucky

**Affiliations:** Department of Cell Biology, Neurobiology and Anatomy, Medical College of Wisconsin, Milwaukee, Wisconsin, United States of America; University of Kentucky Medical Center, United States of America

## Abstract

Subpopulations of somatosensory neurons are characterized by functional properties and expression of receptor proteins and surface markers. CGRP expression and IB4-binding are commonly used to define peptidergic and non-peptidergic subpopulations. TRPA1 is a polymodal, plasma membrane ion channel that contributes to mechanical and cold hypersensitivity during tissue injury, making it a key target for pain therapeutics. Some studies have shown that TRPA1 is predominantly expressed by peptidergic sensory neurons, but others indicate that TRPA1 is expressed extensively within non-peptidergic, IB4-binding neurons. We used FURA-2 calcium imaging to define the functional distribution of TRPA1 among peptidergic and non-peptidergic adult mouse (C57BL/6J) DRG neurons. Approximately 80% of all small-diameter (<27 µm) neurons from lumbar 1–6 DRGs that responded to TRPA1 agonists allyl isothiocyanate (AITC; 79%) or cinnamaldehyde (84%) were IB4-positive. Retrograde labeling via plantar hind paw injection of WGA-Alexafluor594 showed similarly that most (81%) cutaneous neurons responding to TRPA1 agonists were IB4-positive. Additionally, we cultured DRG neurons from a novel CGRP-GFP mouse where GFP expression is driven by the CGRPα promoter, enabling identification of CGRP-expressing live neurons. Interestingly, 78% of TRPA1-responsive neurons were CGRP-negative. Co-labeling with IB4 revealed that the majority (66%) of TRPA1 agonist responders were IB4-positive but CGRP-negative. Among TRPA1-null DRGs, few small neurons (2–4%) responded to either TRPA1 agonist, indicating that both cinnamaldehyde and AITC specifically target TRPA1. Additionally, few large neurons (≥27 µm diameter) responded to AITC (6%) or cinnamaldehyde (4%), confirming that most large-diameter somata lack functional TRPA1. Comparison of mouse and rat DRGs showed that the majority of TRPA1-responsive neurons in both species were IB4-positive. Together, these data demonstrate that TRPA1 is functionally expressed primarily in the IB4-positive, CGRP-negative subpopulation of small lumbar DRG neurons from rodents. Thus, IB4 binding is a better indicator than neuropeptides for TRPA1 expression.

## Introduction

Sensory nerve terminals detect peripheral stimuli in order to discern touch, temperature and pain. These neurons make up a considerably heterogeneous population that can be classified into subgroups based on peripheral targets, functional properties and central projections[1]–[3]. Larger-diameter neurons (≥27 um) tend to have myelinated axons, as with Aβ and Aδ fibers *in vivo*, whereas smaller-diameter neurons (<27 µm) tend to have unmyelinated, C fiber axons *in vivo* [4]. Calcitonin Gene-Related Peptide (CGRP) expression and isolectin B4 (IB4) binding are two common histochemical markers used to define subpopulations of small-diameter neurons[1]–[4]. CGRP is expressed by peptidergic sensory neurons[3]–[6], whereas IB4 binds to α-D-galactose carbohydrate residues typically expressed on the plasma membrane of non-peptidergic neurons[4], [7]–[9]. In peripheral skin targets, IB4-binding neurons terminate superficially between keratinocytes within the epidermis, whereas peptidergic neurons terminate in deeper layers of the epidermis and dermis [10]. Centrally, peptidergic neurons terminate in the outer laminae (I and outer II) of the spinal dorsal horn and target spinal neurons that transmit nociceptive information to the thalamus or brainstem nuclei, regions that mediate the sensory discriminative aspects of pain[10]–[16]. In contrast, the non-peptidergic, IB4-binding population primarily terminates on interneurons within the inner lamina II of the spinal cord and target interneurons that express PKCγ[10], [10]–[14], [17]. Rostrally, the input from IB4-binding neurons ultimately projects to brain areas including the amygdala and hypothalamus, regions involved in affective components of pain [15]. Therefore, it is reasonable to hypothesize that specific receptors that transduce environmental or endogenous stimuli would distribute preferentially between peptidergic and non-peptidergic subpopulations in order to provide selective input to these diverse neural pain pathways.

The Transient Receptor Potential Ankyrin 1 (TRPA1) channel has been the focus of intense interest for its role in inflammatory nociception[18]–[21] and its potential function in transduction of mechanical and cold signals[20], [22]–[25]. Previous investigations of the distribution of TRPA1 among peptidergic and non-peptidergic (IB4-positive) neurons have largely employed *in situ* hybridization and immunohistochemical techniques, which have produced disparate results[18], [21], [26]–[28]. Some studies indicate greater TRPA1 expression in peptidergic, IB4-negative neurons [18], [21], [26], [27], while others found more TRPA1 in non-peptidergic, IB4-binding neurons[27]–[29]. For example, Story and colleagues [26] found TRPA1 mRNA in 3.6% of dorsal root ganglia (DRG) neurons from adult rat, and a majority of these TRPA1-positive cells expressed CGRP. On the other hand, Caspani and colleagues [27] reported that 28% of lumbar 3–6 DRG neurons from adult mouse contain TRPA1 mRNA but only 2–3% of these neurons were CGRP-positive. Discrepancies between studies may have resulted from the inherent limitations of *in situ* hybridization and immunohistochemistry techniques. For *in situ* hybridization, the presence of mRNA does not always accurately predict that the respective protein will be expressed, as RNA can have a high turnover rate and become degraded prior to translation. Immunohistochemistry can sometimes lead to false-positives due to non-specific binding of antibodies or false-negatives due to lower sensitivity of antibodies. Further, receptors might be retained in internal organelles and not functionally expressed at the cell membrane, as has been shown for TRPA1 [30]. The sensitivity of both mRNA and antibody staining approaches depends on the thresholds established for positive versus negative cells.

Calcium imaging combined with live cell markers is a useful approach to determine functional expression of receptors. However, inconsistent results have also been reported for the TRPA1 agonists, allyl isothiocyanate (AITC) and cinnamaldehyde (CINN). Investigators report rates as low as 3–7% [19], [31] to as high as 30% [32] for response to 100 µM CINN. Reports for AITC vary in kind, with 18 to 45% of neurons responding to 50 µM AITC [27], [31]. The discrepant results may be due to the wide range of agonists concentrations and duration of application used in these studies [19], [21], [21,32], the use of different species (mouse versus rat), or the duration neurons are maintained in culture.

Therefore, we set out to resolve the discrepancies in functional TRPA1 expression in identified populations of mouse and rat DRG neurons by using ratiometric calcium imaging and internally consistent parameters. We used lumbar 1–6 DRG ganglia because somata within these DRGs project to skin areas typically probed by behavior assays, such as the plantar hind paw. For both mouse and rat, we used similar isolation protocols, culture duration and media. We did not routinely add exogenous growth factors to the media because adult DRG neurons do not require growth factors for survival [33], and growth factors, such as nerve growth factor (NGF), have been shown to increase the functional expression of TRP channels, including TRPA1 [34], [35]. We identified populations of C fiber-type, small-diameter neurons in live cultures by using IB4-FITC as well as a novel CGRP-GFP mouse where GFP is expressed in CGRPα-expressing neurons. Under these conditions, functional TRPA1 is predominantly found in small-diameter neurons that are IB4-positive and CGRP-negative.

## Materials and Methods

### Materials

Cinnamaldehyde (CINN) and allyl isothiocyanate (AITC) were purchased from Sigma. The same lot was used throughout all experiments. Stock solutions of 100 mM AITC and CINN were made in ethanol and working solutions were prepared in extracellular buffer every 4–5 hours during experiments. Cells were superfused with CINN for 3 min or with AITC for 1 min.

### Animals

Unless otherwise specified, experiments were conducted on male C57BL/6J mice, ages 2–4 months (purchased from Jackson Laboratories). One experiment utilized TRPA1^−/−^ (KO) mice of both sexes (2–12 months old) in which the exons essential for the *Trpa1* gene function were deleted [23]. These TRPA1 KO mice were back-crossed to the C57BL/6J background for over 10 generations. Other experiments utilized CGRP-GFP^+/−^ (CGRP-GFP) mice, a gift from Mark Zylka [36] (males, 3–4 months old), and these mice were also created on a C57BL/6J background. Male Sprague-Dawley rats were purchased from Jackson Laboratories, ages 3–6 months old. All experimental procedures were approved by the Institutional Animal Care and Use Committee of the Medical College of Wisconsin.

### DRG Culture

Mice were briefly anesthetized with isoflurane (Midwest Veterinary Supply) via inhalation and euthanized by decapitation. Lumbar (L) dorsal root ganglia (DRG) 1–6 were isolated bilaterally, unless otherwise specified, and placed into 1 ml Hank’s Balanced Salt Solution (Gibco). After DRG extraction, 1 ml HBSS was replaced with Dulbecco’s Modified Eagle’s Medium/Ham’s nutrient mixture F-12 (DMEM/Hams-F12; Gibco). The ganglia were incubated at 37°C and 5% CO_2_ with 1 mg/ml collagenase Type IV (Sigma) for 40 min, followed by incubation with 0.05% trypsin (Sigma) for 45 min. We used the same protocol for culturing rat DRG neurons except that 2 mg/ml collagenase Type IV (Sigma) was used. Ganglia were washed and resuspended in complete cell medium (see below), then dissociated into single somata via trituration through a P200 pipette tip. The neurons were plated onto laminin-coated glass coverslips and incubated for 2 hours at 37°C and 5% CO_2_ to allow adherence. Coverslips were then flooded with complete cell medium consisting of DMEM/Hams-F12, 10% heat-inactivated horse serum, 2 mM L-glutamine, 0.8% D-glucose, 100 units penicillin and 100 µg/ml streptomycin. Unless noted, no exogenous growth factors were added. For experiments that indicate the inclusion of nerve growth factor (NGF) in the growth media, 100 ng/ml NGF was added to the complete media and incubated with the cells overnight. Most calcium imaging experiments were performed 18–24 hr after cells were plated, with the exception of the experiments specifically conducted at earlier time points (4.5–8.5 hrs and 10.5–14.5 hrs) as noted.

### Retrograde Labeling of Cutaneous Neurons

The medial center aspect of the plantar hind paw of C57BL/6J male mice was injected with 20 µL 1% WGA-Alexafluor594 in sterile saline (Invitrogen). One week after injection, the ipsilateral lumbar 3–5 DRG neurons were cultured as described above. Our prior studies show that in C57BL/6J mice, virtually all labeling from medial plantar hind paw injections occurs in L3-5 DRG ganglia [37]. Cells that fluoresced clearly above background fluorescence levels were targeted for calcium imaging experiments.

### Calcium Imaging and Analysis

Calcium imaging was performed using dual-wavelength fluorescent calcium indicator FURA-2AM (Invitrogen). Isolated DRG neurons were loaded with 2.5 µL/ml FURA-2AM in extracellular buffer containing 2% BSA for 45 min at room temperature, followed by a 30 min wash in extracellular buffer. The extracellular buffer was composed of (in mM): 150 NaCl, 10 HEPES, 8 glucose, 5.6 KCl, 2 CaCl_2_ and 1 MgCl_2_ (pH 7.4, 320±3 mOsm). Coverslips with loaded cells were mounted onto a perfusion chamber and superfused with buffer at a constant rate of 6 ml/min using an AutoMate pressurized perfusion system. A 4-way manifold system with a 2 cm bath inlet provided immediate release of buffer and chemical agents into the superfusion chamber. This superfusion system allowed no dead space and minimized air bubbles in the lines. Identical tubing was used for each input line. Experiments were conducted at room temperature (23°±1°C). Fluorescence images were captured with a cooled CCD camera (CoolSNAP FX; Photometrics, Tucson, AZ). Metafluor imaging software was utilized in order to detect and analyze intracellular calcium changes throughout the experiment (Molecular Devices, Sunnyvale, CA). A ≥20% increase in intracellular calcium from baseline was considered a response. All neurons were tested with only one concentration of one agonist. At the end of each protocol, 50 mM KCl solution was applied to depolarize neurons, thereby allowing for identification of viable neurons from non-neuronal cells or non-functioning neurons. Neuronal viability of non-responsive cells was confirmed by response to 50 mM KCl. For mouse, neurons were considered “small” if the average somata diameter was less than 27 µm. Rat neurons were considered “small” if the average somata diameter was less than 30 µm.

### IB4 Staining

Upon completion of imaging protocol, cells were incubated with 10 µg/ml isolectin B4 conjugated to fluorescein isothiocyanate (IB4-FITC; Sigma) for 10 min and then washed with extracellular buffer and visualized using FITC filters. Neurons were considered to be IB4-positive neurons if they had a complete ring of FITC stain around the soma perimeter.

### Determination of CGRP-GFP-positive Cells

Small diameter cells from the CGRP-GFP^−/+^ mice were separated into CGRP-positive and –negative subgroups. Cells were deemed CGRP-positive if their GFP fluorescence was ≥1 standard deviation above background fluorescence. Background fluorescence was determined by fluorescence levels of wild-type neurons from the CGRP-GFP mouse line that did not express GFP (fluorescence due to autofluorescence only).

## Results

### TRPA1 Agonists AITC and CINN Elicit Calcium Responses with Different Latencies in Adult Mouse DRG Neurons

**Figure 1:**
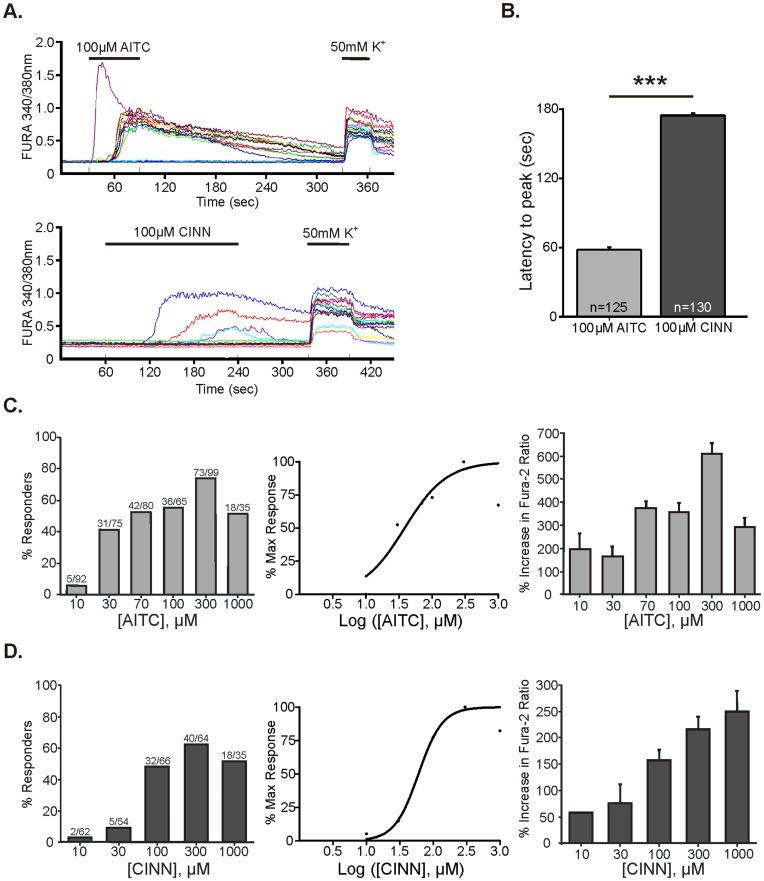
TRPA1 agonists allyl isothiocyante and cinnamaldehyde elicit calcium responses with different latencies in DRG neurons. A. Representative traces of individual, dissociated lumbar 1–6 dorsal root ganglia (DRG) neurons from C57BL/6J wild-type mice during FURA-2 calcium imaging. Separate neurons were tested with 100 µM allyl isothiocyanate (AITC; 1 min; top trace) or cinnamaldehyde (CINN; 3 min; bottom trace) at 6 ml/min. Note that the latency to peak amplitude for CINN responses was longer than that for AITC, whereas responses to 50 mM K+ (30 s) occurred almost immediately after the start of superfusion. B. Average latency from the start of superfusion to maximum amplitude of response for AITC and CINN (3 culture preparations from 5–6 animals for each group). The latency between onset of superfusion and peak response for CINN was significantly longer than that for AITC (p<0.0001; t-test). C–D. Concentration-response curves for AITC (C) and CINN (D) for percentage of responders (left two panels) and peak amplitude of calcium responses (right panel). Neurons that exhibited a ≥20% increase in FURA ratio from baseline during agonist superfusion were considered “responsive” (3 cultures from 5–6 animals for each group; AITC: 446 total neurons; CINN: 281 total neurons). All neurons were tested with only one concentration of one agonist. For percentage of responders, the EC50 for AITC was 38.5 µM and that for CINN was 60.2 µM (both calculated from 10–1000 µM data). For peak response amplitude, the EC50 for AITC was 115.3 µM (calculated from 10–300 µM data) and that for CINN was 97.5 µM (calculated from 10–1000 µM data).

Allyl isothiocyanate (AITC) and cinnamaldehyde (CINN) are commonly-used exogenous agonists of TRPA1 [19], [21], [21], [26,32]. Figure 1A shows responses of mouse lumbar 1–6 dorsal root ganglia (DRG) neurons to AITC or CINN (both 100 µM). AITC induced an increase in intracellular calcium, reaching a peak within 58±2 sec from the onset of perfusion onto cells (Fig. 1B). Alternatively, CINN-evoked excitation was preceded by a longer delay with calcium peak occurring after 175±2 sec (Fig. 1B). This difference in response latency was not due to imperfections in our superfusion system since 50 mM potassium induced an immediate response for every application (Fig. 1A). The superfusion rate for all chemicals was consistently 6 ml/min, and switching stimuli to buffer only did not elicit a change in intracellular calcium, indicating that the stimulus-induced calcium increases were not due to mechanical stimulation. Responses to 50 mM potassium also served as a positive control for neuronal viability. Due to the differences in latency to response for CINN and AITC, we superfused cells with CINN for 3 min and with AITC for 1 min (both 100 µM) to ensure we included all responders in our analysis.

We performed concentration-response curves for each agonist. A ≥20% increase in FURA 340/380 ratio over baseline during agonist superfusion was considered a response, and neurons were tested with one concentration of only one agonist as depicted in Figure 1A. For percentage of neurons activated, the EC50 for AITC was 38.5 µM whereas that for CINN was 60.2 µM. (Fig. 1C, D). Both compounds activated a maximum percentage of neurons at approximately 300 µM. We defined the magnitude of response as the amplitude of the increase in Fura-2 ratio from baseline evoked by each stimulus. For magnitude of response, the EC50 for AITC was 115.3 µM, whereas that for CINN was 97.5 µM.

**Figure 2:**
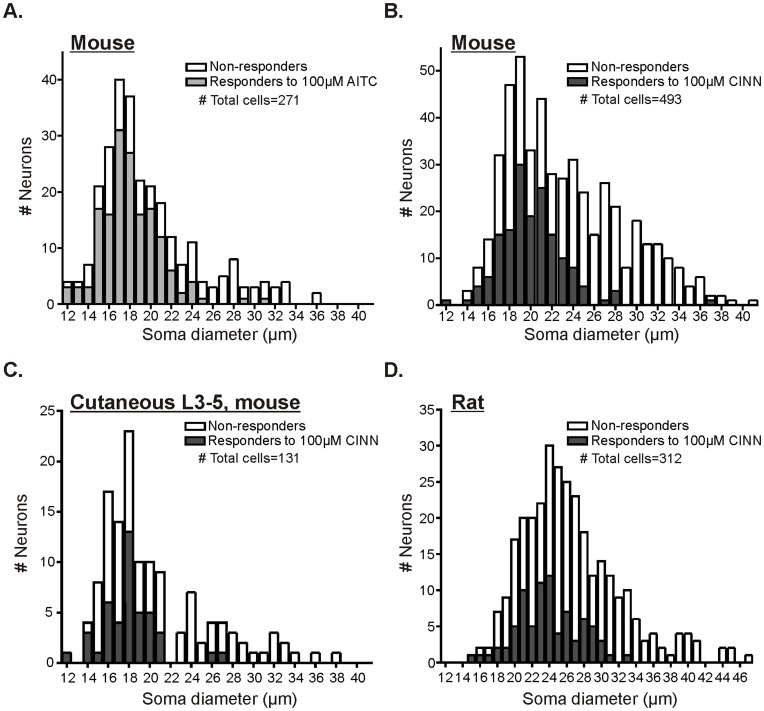
Cell size distributions of adult mouse and rat DRG neurons responding to TRPA1 agonists. Distribution of somata diameters (1 µm bins) of lumbar DRG neurons in culture preparations from mouse or rat. Number of neurons responding to AITC or CINN (both 100 µM) are shown in grey bar portion, non-responsive neurons are shown in white bar portion, and total cells in each size bin are reflected by the top of the bar (sum of the grey and white bars). A. Mouse lumbar 1–6 DRG neurons tested with 100 µM AITC (3 cultures from 6 animals; 271 total neurons; 160 responders; 59% responders). B. Mouse lumbar 1–6 DRG neurons tested with 100 µM CINN (7 cultures from 7 animals; 493 total neurons; 160 responders; 32% responders). C. Mouse neurons labeled via retrograde tracer injected into the medial plantar hind paw. All neurons were taken from lumbar 3–5 DRGs ipsilateral to injection. Only labeled neurons were used for recordings. Thus all neurons in the graph were labeled from the hind paw plantar skin. Neurons were tested with 100 µM CINN (5 cultures from 10 animals; 131 total neurons; 43 responders; 33% responders). D. Rat lumbar 1–6 DRG neurons tested with 100 µM CINN (3 cultures from 3 animals; 312 total neurons; 80 responders; 26% responders).

Next we determined the size distribution of the somata of lumbar DRG neurons that respond to AITC and CINN. While a number of studies indicate that large neurons do not express TRPA1 immunoreactivity[24], [28], [38]–[40], prior data from our laboratory has shown that the terminals of some cutaneous Aβ fiber low-threshold neurons express TRPA1 protein, and mechanical responsiveness of several types of cutaneous Aβ and Aδ fibers are altered in global TRPA1-null mice [41]. However, here we found that very few neurons greater than 27 µm responded to either AITC (6.3%; 2/32) or CINN (3.6%; 5/140; Figs. 2A, B) and therefore, we focused the rest of the experiments on small-diameter (<27 µm) neurons.

To determine whether AITC and CINN at a near maximal concentration (both 100 µM) are specific agonists for TRPA1, we tested responses in small-diameter lumbar DRG neurons from global TRPA1-null mice [23]. AITC evoked calcium increases in only 4.3% (7/162) of small neurons, whereas CINN elicited responses in just 2% (4/193) of the neurons (Fisher’s exact, p = 0.2386, n.s.). These data indicate that both AITC and CINN at 100 µM selectively target the TRPA1 channel protein on isolated DRG somata. Since CINN elicited slightly fewer responses in TRPA1-null neurons, we used CINN for subsequent experiments where only one TRPA1 agonist was utilized.

### TRPA1 is Functionally Expressed Predominantly by Non-peptidergic, IB4-binding Neurons

**Figure 3:**
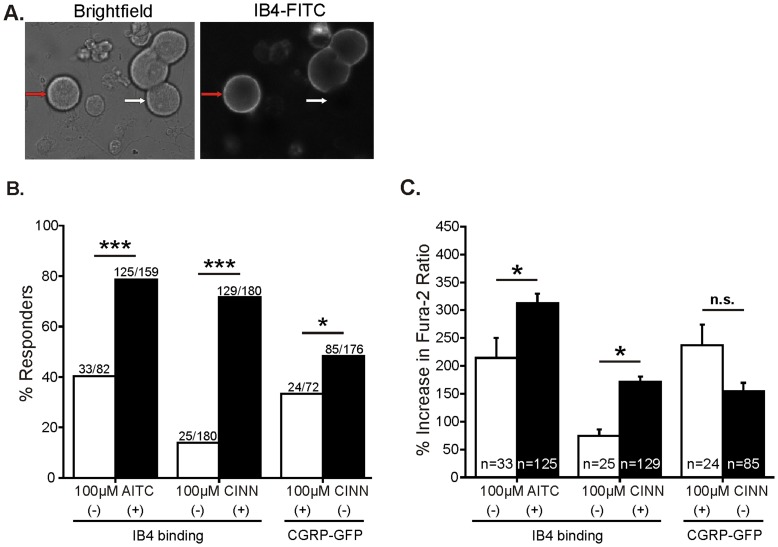
TRPA1 is functionally expressed predominantly by IB4-positive and CGRP-negative neurons. A. Representative brightfield (left) and FITC (right) images of lumbar 1–6 DRG neurons from C57BL/6J wild-type mouse stained with IB4-FITC (60x objective). IB4-positive neurons were defined by a halo of FITC-labeling around the entire perimeter of the somata of small-diameter (<27 µm) neurons. The red arrow indicates an IB4-positive neuron, whereas the white arrow indicates an IB4-negative neuron. B. Percentage of small-diameter neurons responding to TRPA1 agonists (all 100 µM) defined by IB4-binding or CGRP expression. A greater percentage of IB4-positive neurons responded to TRPA1 agonists than the percentage of IB4-negative neurons (overall effect: Chi square p<0.0001; ***p<0.0001). Likewise, a greater percentage of CGRP-negative neurons responded to CINN than CGRP-positive neurons (*p<0.0350 Fisher’s exact). CGRP data were generated from 3 animals in 2 cultures. C. Amplitude of responses to TRPA1 agonists (all 100 µM) of small-diameter DRG neurons defined by IB4-binding or CGRP expression. IB4-positive neurons responded with a greater peak average amplitude than IB4-negative neurons (overall effect: ANOVA p<0.0001; *p<0.05, Tukey). There was no difference between CGRP-positive and CGRP-negative peak amplitudes.

To determine which populations of small-diameter neurons typically respond to TRPA1 agonists, we incubated lumbar DRG neurons from adult mouse with IB4 conjugated to FITC (IB4-FITC) after exposure to AITC or CINN. Examples of neurons stained with IB4-FITC are shown in Figure 3A. IB4-positive neurons were identified by a complete halo of FITC fluorescence around the perimeter of the soma. Approximately 50% of all small neurons from mouse lumbar ganglia stained positively for IB4 (56%; 339/601). Among all small neurons tested, 66% (158/241) responded to 100 µM AITC and 42% (155/366) responded to 100 µM CINN. Next, we sorted the small-diameter neurons into IB4-positive and IB4-negative. Among all IB4-positive neurons, 79% (125/159) responded to AITC and 72% (129/180) responded to CINN (Fig. 3B). Conversely, among all IB4 negative neurons, only 40% (33/82) responded to AITC and 14% (25/180) responded to CINN (Fig. 3B). Overall, approximately 80% of neurons responding to either AITC (79%; 125/158) or CINN (84%; 129/154) were IB4 positive. In addition, the IB4-positive responsive neurons exhibited a greater peak magnitude to both AITC and CINN than did the IB4-negative responsive neurons (Fig. 3C). These findings were consistent across ten independent experiments (ten separate animals and culture preparations). Thus, TRPA1 appears to be functionally expressed mainly by IB4-positive small-diameter neurons in adult mice.

**Figure 4:**
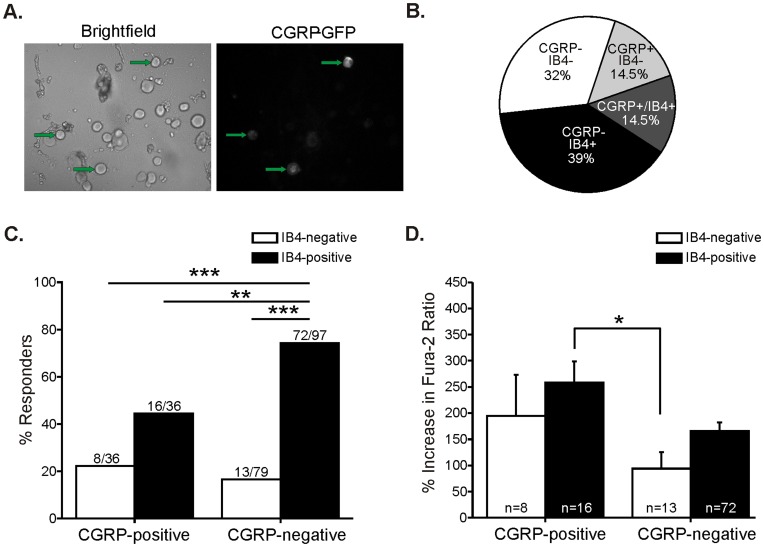
TRPA1 is functionally expressed mainly by neurons that are both IB4-positive and CGRP-negative. A. Representative brightfield (left) and FITC (right) images of lumbar 1–6 DRG neurons from the CGRP-GFP^+/−^ mouse strain (20x objective). CGRP-positive neurons were identified by GFP fluorescence levels that were three standard deviations above the average autofluorescence levels of DRG neurons from CGRP-GFP^+/+^ wild-type mice. The green arrows indicate CGRP-positive neurons. B. Distribution of both CGRP expression and IB4 binding among all small-diameter lumbar 1–6 DRG neurons (248 total neurons). A total of 29% of small neurons were CGRP positive. Note that 50% of these (14.5%) were also IB4-positive. C. Percentage of small diameter neurons responding to 100 µM CINN defined by IB4 *and* CGRP labeling. A greater percentage of IB4-positive/CGRP-negative neurons responded to CINN than the percentage of responders with other staining combinations (overall effect: Chi square p<0.0001; ***p<0.0001, **p = 0.0019, Fisher’s exact for 2-group comparisons). The majority of responders (66%; 72/109) were IB4-positive *and* CGRP-negative. D. Amplitude of responses to 100 µM CINN grouped by CGRP and IB4 labeling. The responses of IB4-positive/CGRP-positive were significantly greater than those for IB4-negative/CGRP-negative neurons (overall effect: ANOVA p = 0.0250; *p<0.05 Tukey post hoc test).

To confirm our findings using an alternate label, we used CGRP as a marker for the peptidergic subgroup in neurons cultured from a novel CGRP-GFP mouse line in which GFP expression is driven by the CGRPα promoter [36] (Fig. 4A). In this mouse strain, the majority (89%) of all neurons that were immunoreactive for a CGRP antibody (which recognizes both α and β CGRP isoforms) were CGRPα-GFP-positive [36]. Likewise, the majority (68%) of CGRPα-GFP-positive neurons bound the CGRP antibody [36]. The finding that not all CGRPα-GFP-positive neurons expressed CGRP immunoreactivity was likely due to greater sensitivity and detection of the GFP marker than the CGRP antibody [36]. First we found that there was no difference between the overall percentage of small neurons that responded to 100 µM CINN or mean amplitude of responses between neurons from CGRP-GFP mice (44%; 109/248; mean response amplitude 173±14%) compared to those from wild-type (C57BL/6J) mice (42%; 155/366; mean response amplitude 155±9%), indicating that the mouse strains, which are both on a C57BL/6J background, express similar levels of TRPA1 at the lumbar DRG level. Significantly more CGRP-negative neurons (48%; 85/176) responded to CINN compared to CGRP-positive neurons (33%; 24/72; Fig. 3B). Among small neurons that responded to CINN, 78% (85/109) were CGRP-negative and only 22% (24/109) were CGRP-positive. However, there was no difference in the average amplitude of response to CINN in CGRP-negative and CGRP-positive responders (Fig. 3C). The CGRP-positive responders exhibited a trend for larger responses, but this was not significantly different.

Next, to determine the overlap in IB4 binding and CGRP expression in small neurons, we used IB4 staining with a red fluorophore (IB4-AlexaFluor594) together with the CGRP-GFP mouse neurons. Among all small-diameter lumbar DRG neurons, a total of 29% (72/248) were CGRP-positive. Surprisingly, among the CGRP-positive neurons, 50% (36/72) were co-labeled for IB4 (Fig. 4B). There was a significantly greater percentage of IB4-positive/CGRP-negative neurons responding to CINN than neurons with other staining combinations (Fig. 4C). In fact, the majority of neurons that responded to CINN were IB4-positive and CGRP-negative (66%; 72/109). IB4-negative/CGRP-negative neurons exhibited significantly smaller response amplitudes to CINN than IB4-positive/CGRP-positive neurons (Fig. 3C). Togther, these data indicate that TRPA1 is functionally expressed primarily by IB4-positive/CGRP-negative neurons in mouse. These data also suggest that IB4 staining is a better predictor of neurons that are likely to respond to TRPA1 agonists than is CGRP expression.

### Plantar Skin-projecting Neurons Express TRPA1 Primarily in IB4-positive Neurons

Lumbar DRG neurons are considerably heterogeneous with respect to their peripheral target projections, which include skin, muscle, bone, tendons and visceral organs. Many of the non-peptidergic, IB4-binding or MrgprD-positive neurons innervate superficial skin [3], [11], [12], [14], [42]. Because the plantar skin of the hind paw is a common region examined in behavioral assays and is an area used for *ex vivo* skin-nerve electrophysiological preparations, we retrograde-labeled DRG somata that project to this skin region with WGA-Alexafluor594. After 7 days, neurons from ipsilateral lumbar 3–5 ganglia were dissociated and cultured. Neurons that were strongly labeled with distinct, bright red fluorescence were imaged. The mean diameter of retrograde-labeled (cutaneous) neurons was smaller (20.8±0.5 µm) than those of mixed lumbar 1–6 DRG cultures (23.7±0.2 µm; compare Figs. 2B, C; t-test, p<0.0001). The difference in cell sizes was expected since most afferents innervating the superficial skin correspond to non-peptidergic (IB4-positive), small-diameter neurons [3], [9], [11], [12], [43]. Similar to lumbar DRG neurons with mixed peripheral targets, 38% (42/112) of all small-diameter cutaneous neurons responded to 100 µM CINN. Additionally, the mean response amplitudes were similar between cutaneous (159.4±16.9; n = 42) and mixed target responders (155.7±8.7; n = 154). As in our results with mixed target DRG cultures, significantly more IB4-positive cutaneous neurons (50%, 34/68) responded to CINN than IB4-negative neurons (18%, 8/44; Fisher’s exact, p = 0.0007). Out of all cutaneous neurons responding to CINN, 81% (34/42) were IB4-positive. IB4-positive cutaneous neurons responded with an average amplitude (171±19%) that was not different than IB4-negative neurons (110±33%; t-test, p = 0.1588). These data indicate that TRPA1 is functionally present in neurons that innervate plantar hind paw skin and, as with DRG somata with mixed peripheral targets, most cutaneous neurons that express functional TRPA1 are IB4-positive.

### The Pattern of TRPA1 Expression Differs between Mouse and Rat DRG Neurons

**Figure 5:**
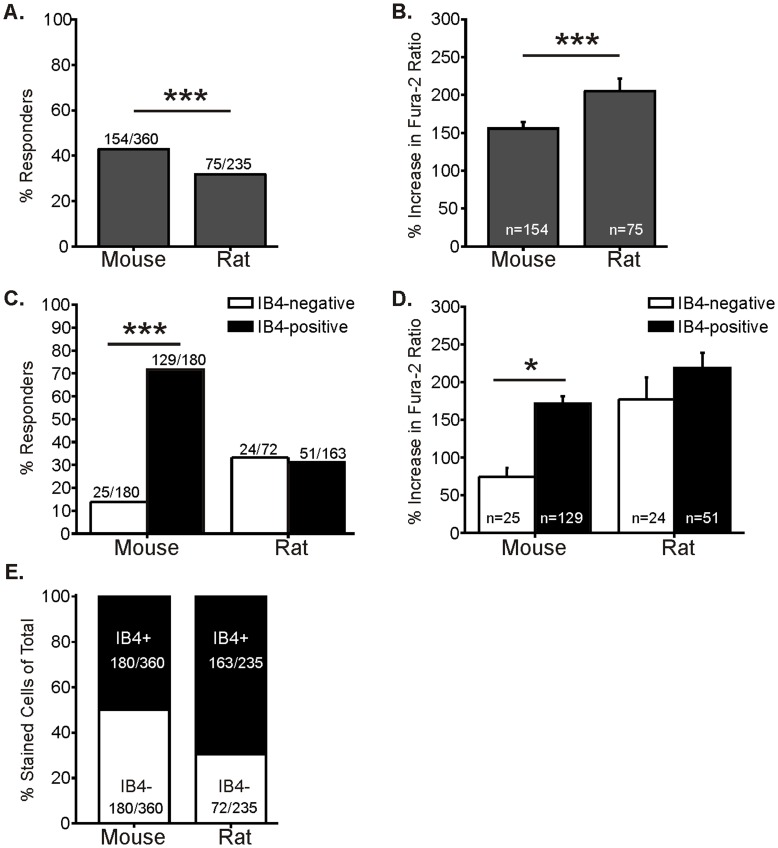
The pattern of functional TRPA1 expression differs between mouse and rat DRG neurons. A. Percentage of small-diameter mouse and rat DRG neurons responding to 100 µM CINN. Small-diameter neurons for mouse were defined as less than 27 µm, whereas those for rat were defined as less than 30 µm in soma diameter. Significantly more small-diameter mouse neurons responded to CINN than rat neurons (***p = 0.0096, Fisher’s exact). B. Peak amplitude of responses for mouse and rat small-diameter neurons to 100 µM CINN. Rat neurons had a greater response amplitude to CINN than did mouse neurons (***p = 0.0039, t-test). C. Percentage of mouse and rat neurons responding to 100 µM CINN defined by IB4 staining. For mouse, significantly more IB4-positive neurons responded than IB4-negative neurons (left bars). However, for rat there was no difference between IB4-positive and IB4-negative neurons (overall effect: Chi square p<0.0001; ***p<0.0001, Fisher’s exact). Nonetheless, among the rat neurons that responded to CINN, the majority were IB4-positive (68%; 51/75). The mouse data set (left two bars) is the same data as previously shown in Fig 3B (middle two bars). D. Amplitude of responses to 100 µM CINN of mouse and rat neurons defined by IB4 binding. In mouse, the IB4-positive neurons responded with greater amplitudes than IB4-negative neurons. In contrast to mouse, there was no difference between the amplitudes of IB4-positive and IB4-negative neurons from rat (overall effect: ANOVA p<0.0001; *p<0.01, Tukey post hoc test). The mouse data (left two bars) is the same as shown in Fig 3C (middle two bars). E. Distribution of IB4 binding among all small-diameter neurons from mouse and rat. Significantly more neurons were IB4-positive in rat than in mouse (p<0.0001, Fisher’s exact).

A frequent question is whether rat and mouse DRG neurons are similar. An optimal approach to compare species is to use identical techniques in the same laboratory to determine whether there are inter-species differences in the distribution of TRPA1 in sensory neurons. Therefore, we conducted calcium imaging experiments on lumbar 1–6 DRG neurons isolated from rat. The same culturing techniques and media were used, except twice the concentration of collagenase was used for chemical dissociation for rat DRGs than for mice (see Methods). We found a number of differences between rat and mouse sensory neurons. First, as expected, the distribution of soma diameters for all lumbar DRG neurons is larger in rat than mouse (Figs. 2D, B). The range of cell sizes for rat was 15–47 µm (median: 25.8 µm), whereas the size range for mouse neurons was 12–41 µm (median: 22.5 µm). Further, the size of neurons responding to CINN was significantly larger in rat than mouse (Figs. 2D, B). The rat neurons that responded to CINN had a mean soma diameter of 24.4±0.4 µm (median: 24 µm; n = 80), whereas the mean diameter of CINN responders from mouse was 20.6±0.2 µm (median: 20.4 µm; n = 160; t-test, p<0.0001). Based on the size of CINN responders in rat, we considered small-diameter neurons to be those less than 30 µm in diameter, compared to 27 µm in mouse. Mouse and rat neuronal populations had different percentages of responders and mean response amplitudes to CINN (Figs. 5A, B). Fewer total small-diameter neurons from rat responded to CINN, but the responsive neurons in rat had greater response amplitudes than those from mouse (Figs. 5A, B). In contrast to mouse where a larger percentage of IB4-positive neurons functionally express TRPA1 than the percentage of IB4-negative neurons, the percentage of IB4-positive and IB4-negative neurons responding to CINN was similar for rat (Fig. 5C). However, of the responding neurons in rat, the majority (68%; 51/75) were IB4-positive. The peak magnitude response for IB4-positive and IB4-negative neurons in rat was also similar (Fig. 5D). Interestingly, these differences between mouse and rat did not appear to result from fewer small rat neurons labeled with IB4 since significantly more small neurons from rat were IB4-positive compared to mouse (Fig. 5E; p<0.0001 Fisher’s exact). Despite these details, the overall data indicate that most neurons that functionally express TRPA1 are IB4-positive in rat as in mice.

### TRPA1 Function and Distribution Changes with Duration in Culture

**Figure 6:**
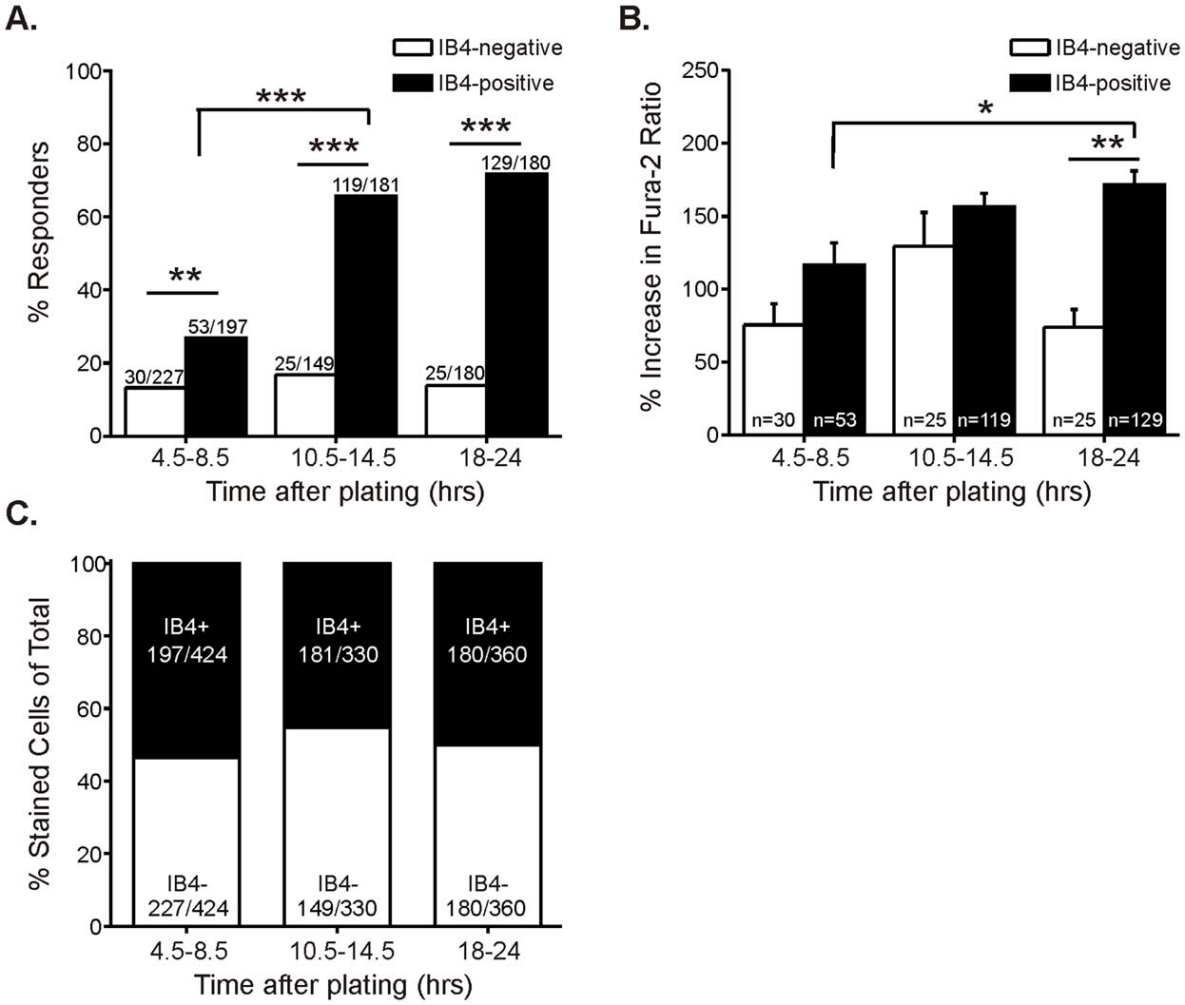
TRPA1 function in IB4-positive neurons increases with duration in culture. A. Percentage of small-diameter L1-6 DRG neurons from adult mouse responding to 100 µM CINN defined by IB4 binding and duration between plating and imaging cells (5–7 cultures prepared from 5–7 animals). The percentage of IB4-positive responders significantly increased between the 4.5–8.5 hr and the 10.5–14.5 hr time points after plating (overall effect: Chi square p<0.0001; ***p<0.0001, Fisher’s exact). At the earliest time point tested (4.5–8.5 hrs), although there was a smaller percentage of IB4-positive neurons responding to CINN than at the later time points, there were still significantly more IB4-positive than IB4-negative neurons responding at this time (**p = 0.0005, Fisher’s exact). In contrast, there was no difference in IB4-negative responders across any of the time points. The data set for 18–24 hrs (right two bars) is the same as that shown in Fig 3B (middle two bars). B. Average amplitude of responses to 100 µM CINN defined by IB4 binding and duration between plating and imaging neurons. The amplitude of responses for IB4-positive neurons increases between 8.5 and 18 hrs (overall effect: ANOVA p<0.0001; *p<0.05, Tukey post hoc test). There was no overall difference in the amplitude of responses for IB4-negative responders. C. Distribution of IB4 staining among all small-diameter neurons defined by duration between plating and imaging cells. There was no significant change in IB4 binding across any of the time points tested (Chi square p = 0.0735). The data set for 18–23 hrs (right bar) is the same as shown in Fig 5E (left bar).

Time in culture is another factor that may potentially affect response to TRPA1 agonists. We therefore recorded from mouse neurons at 4.5–8.5 hr, 10.5–14.5 hr, and 18–24 hr after plating. As the culture duration progressed, we found that a greater percentage of small-diameter neurons responded to CINN. The increase in responders over time occurred specifically among IB4-positive neurons, and functional TRPA1 levels stabilized by 10.5 hr after the neurons were plated (Fig. 6A). Although there were fewer IB4-positive neurons responding to CINN at the earliest time point (4.5–8.5 hr), we still found significantly more IB4-positive neurons with functional TRPA1 than IB4-negative neurons (Fig. 6A). The response amplitudes also increased as culture duration progressed, and this effect was specific to IB4-positive neurons (Fig. 6B). We also observed greater response amplitudes for IB4-positive than for the IB4-negative responders at 18–24 hrs, but not at earlier time points (Fig. 6B). There was no difference in the percentage of neurons that stained positively for IB4 at any time point (Fig. 6C), suggesting that the increase in IB4-positive CINN-responsive neurons is not likely due to changes in IB4-binding.

### Exogenous NGF has No Effect on Distribution of TRPA1 Expression

In all of the previous experiments, we did not use exogenous growth factors in our culture media since nerve growth factor (NGF) can alter responsiveness of a number of TRP channels, including TRPA1 [34], [35], [44], and adult DRG neurons do not require growth factors such as NGF to survive in culture [33]. However, this is a factor that varies between studies and might affect expression of TRPA1. Therefore, we investigated the influence of exogenous NGF (100 ng/ml, overnight) on the functional distribution of TRPA1 *in vitro* (DRGs from 3 mice in 3 separate cultures). We found that significantly more total small neurons cultured with NGF responded to 100 µM CINN (53%; 115/216) than those cultured without NGF (42%; 155/366; Fisher’s exact, p = 0.0126). Response amplitudes, however, were not affected (155.7±8.7% without NGF; 155.6±10.4% with NGF). When grouped by IB4 binding, we found that significantly more IB4-positive neurons (79%; 93/118) responded to CINN than IB4-negative neurons (22%; 22/98) after exposure to NGF. These results were not different than our results without NGF exposure (IB4-positive neurons: 72%; 129/180; IB4-negative neurons: 14%; 25/180). Further, IB4-positive neurons treated with NGF overnight still exhibited greater peak response amplitudes than IB4-negative neurons (IB4-positive: 174.1±11.5% n = 93; IB4-negative: 77.5±15.5% n = 22; t-test p = 0.0002). These responses were not different from untreated neurons (IB4-positive: 171.6±9.5% n = 129; IB4-negative: 74.1±12% n = 25). Therefore, although more neurons functionally express TRPA1 after treatment with exogenous NGF, the distribution of functional TRPA1 is not affected. Even with NGF treatment, more IB4-positive neurons functionally express TRPA1 than IB4-negative neurons.

## Discussion

Here we set out to resolve the contrasting claims regarding the populations of DRG neurons that express functional TRPA1 channels. Using standardized conditions, our results demonstrate that the majority of DRG neurons from mouse that respond to TRPA1 agonists are small-diameter, IB4-positive and CGRP-negative. Similarly in rat, most neurons that functionally express TRPA1 were IB4-positive. Very few large-diameter neurons from either species responded to TRPA1 agonists. In addition, we found that culture duration affects TRPA1 function in that responsiveness to TRPA1 agonists increases specifically in IB4-positive neurons despite the lack of added exogenous growth factors.

### TRPA1 Functional Expression Correlates Extensively with IB4 Binding and Less with CGRP Expression

Contrary to a number of previous studies using *in situ* hybridization and immunochemistry that reported high TRPA1 expression among peptidergic, IB4-negative neurons [18], [21], [26], [27], we consistently found that the majority of small-diameter neurons that respond to TRPA1 agonists are IB4-binding and CGRP-negative. Our results agree with those of Hjerling-Leffler and colleagues [29], Kim and colleagues [28] and Caspani and colleagues [27] who reported high TRPA1 expression in IB4-positive neurons from mouse and rat. Disparities on this matter appear to be due to the markers used in each study. Those studies employing IB4 binding have all found high overlap between TRPA1 and IB4 binding, whereas studies that examined only CGRP or Substance P expression concluded that TRPA1 is expressed by peptidergic neurons. Our study is the first to employ both IB4 binding and CGRP expression in live neurons that are tested with TRPA1 agonists. We find that IB4 binding is a better indicator of TRPA1 expression than is CGRP expression in mouse. Surprisingly, we found that half of the CGRP-positive neurons in mouse bind IB4. Therefore, CGRP and IB4 do not label exclusive populations in mouse lumbar DRGs and caution should be exercised when using IB4 to identify “non-peptidergic” neurons.

As in mice, the majority of TRPA1-responsive neurons in rats were IB4-positive. However, while 80% of IB4-positive mouse neurons responded to CINN, only 30% of IB4-positive neurons from rat responded. This difference was not due to a lower IB4 binding as more neurons were IB4-positive in rat than mouse. The difference is likely due in part to a lower overall responsiveness to TRPA1 agonists in rat.

### IB4 Positive Neurons and TRPA1 both Play Key Roles in Mechanical Pain Behavior

Accumulating evidence suggests that IB4-binding neurons play particular roles in somatosensation and pain. First, in non-injured skin, Mrgprd-positive afferents, which comprise approximately 90% of IB4 positive cutaneous afferents, are critical for transduction of noxious mechanical, but not thermal, stimuli [45]. Second, IB4-binding neurons are especially subject to plasticity after tissue injury. They become sensitized to mechanical stimuli in a chronic model of sickle cell disease pain [46]. Additionally, mechanical hypersensitivity after nerve injury and muscle inflammation depend on IB4-positive nerve fibers [45], [47]. IB4-positive neurons have been shown to play a key role in hyperalgesic priming[48]–[50], whereby the inflammatory mediators NGF and prostaglandin E_2_ (PGE_2_) initiate activation and membrane translocation of PKCε selectively in IB4-positive nociceptors[50]–[54]. Hyperalgesic priming in IB4-positive nociceptors has been proposed to mediate the transition from acute to chronic pain [50], [54]. Our finding that TRPA1 expression increases selectively on IB4-positive neurons with time in culture is consistent with the idea that dissociation of DRGs may simulate a type of “injury” that DRG neurons recover from over time.

The finding that functional TRPA1 is preferentially expressed by IB4-positive neurons suggests that TRPA1 may mediate specific contributions of IB4-positive neurons to mechanical pain. Growing evidence suggests that TRPA1 plays an integral role in both the development and maintenance of inflammatory mechanical hyperalgesia [18], [40], [55], [56], and TRPA1 inhibition at the peripheral terminal reverses the mechanical sensitization of C fibers after inflammation [57]. Thus, the TRPA1-expressing IB4-positive neurons may be particularly important for conveying mechanical pain from skin. TRPA1 is a promising target for therapeutic intervention of inflammatory pain, and therapies targeting TRPA1 on IB4-positive neurons may reduce mechanical hyperalgesia without abolishing normal tactile acuity for patients that suffer from inflammatory pain.
